# The influence of informal social support on risk and prognosis in spinal pain: A systematic review

**DOI:** 10.1016/j.ejpain.2010.09.011

**Published:** 2011-05

**Authors:** Paul Campbell, Gwenllian Wynne-Jones, Kate M Dunn

**Affiliations:** Arthritis Research UK Primary Care Centre, Keele University, United Kingdom

**Keywords:** Social support, Social network, Spinal pain, Review, Biopsychosocial

## Abstract

Spinal pain is very common and has considerable consequences for the individual (e.g. loss of employment, disability) as well as increased health care costs. It is now widely accepted that biological, psychological and social factors impact on spinal pain outcomes. The majority of research on social factors has been employment related, with little attention to the influence of informal social support (e.g. families, friends, social groups). The aim of this review is to investigate whether informal social support is associated with the occurrence and prognosis of spinal pain. Prognosis was considered in a broad sense within the biopsychosocial model inclusive of factors such as pain, function, general and psychological health. A systematic search of eight databases was conducted to search for studies who report findings on informal social support in populations with nonspecific spinal pain (i.e. no defined cause). Seventeen articles were identified and a best evidence synthesis was carried out on the data extracted from the studies. Results show that for cross-sectional designs there was inconclusive evidence of a relationship between social support and pain but moderate evidence of a relationship between social support and patient psychological outcome related to prognosis. Evidence of social support as a factor for risk of occurrence was inconclusive with three studies reporting no significant associations with the remaining two studies reporting weak associations. Evidence of an effect of social support and prognosis revealed inconsistent findings. The variation in findings may reflect ongoing difficulties surrounding the conceptualisation and measurement of informal social support.

## 1 Introduction

Spinal pain is very common in the general population. Three large population studies place a life time prevalence of neck pain at 40–66%, and a life time prevalence of back pain at 60–80% (Papageorgiou et al., 1995, Cote et al., 1998, Leboeuf-Yde et al., 2009). In addition, up to 50% of spinal pain sufferers seek health care in relation to their pain (Picavet and Schouten, 2003) leading to substantial healthcare costs, both direct (e.g. treatment) and indirect (e.g. informal care, loss of earnings, state support) for the individual, health care and society (Dagenais et al., 2008).

It has been broadly accepted that processes involved in risk, prognosis and treatment of spinal pain are complex, and incorporate factors associated with the biopsychosocial model (Waddell, 2004, Turk and Okifuji, 2002). Since the introduction of this model, there has been widespread application within research as well as implementation in treatment guidelines for back pain (e.g. European guidelines, van Tulder et al., 2002).

One area for focus within social influence research is informal social support. Informal social support is defined as support provided outside formal settings (i.e. not workplace, health professional or social service support). It includes support from family, friends and informal groups. Although difficult to conceptualise (Hutchison, 1999), there is broad consensus that four main constructs are thought to encompass the different types of support that can be given (Langford et al., 1997): (1) emotional support (e.g. emotional support in a crisis), (2) instrumental support (e.g. getting help to get to and from hospital), (3) informational support (e.g. receiving advice), (4) appraisal support (e.g. being listened to). These constructs are further moderated by the structural or social network a person may have (i.e. number of persons available) and the perceived satisfaction about the support (Sarason et al., 1983). Two main theoretical hypotheses profess beneficial effects of social support. Firstly social support promotes general good health and protects from getting ill and, secondly, having social support promotes a better recovery from illness. Research on general health has shown a lack of social support led to an increase risk of mortality (Berkman and Syme, 1979, House et al., 1988), and as a significant barrier in a person’s recovery from illnesses (Kroenke et al., 2006, Chronister et al., 2008). However a recent review argues that the direction of research on chronic pain has centred more on biological and psychological aspects and largely overlooked social factors (Blyth et al., 2007). In support, a review of review articles, of studies on back pain, confirm that there are no firm conclusions on social support unrelated to the workplace (Hayden et al., 2009).

In this article the aims are to summarise the evidence of the effect of informal social support on the occurrence and prognosis of nonspecific spinal pain. As prognosis of spinal pain is considered as a multifactorial construct within the biopsychosocial model (Bombardier, 2000, Gatchel et al., 2007), the contribution of informal support to psychological complaints in patients with nonspecific spinal pain will also be reviewed.

## 2 Methods

### 2.1 Search strategy

This review uses a systematic approach to identify and synthesise research within nonspecific spinal pain populations on informal social support. Nonspecific spinal pain populations were targeted as they represent the majority of cases of spinal pain with estimations of up to 95% of patients having uncomplicated (i.e. no serious malignancy or neurologic deficits) for low back pain (Deyo and Phillips, 1996). Various types of social support are considered, including emotional, instrumental, informational, appraisal, network size and perceived satisfaction of support.

The following computerised databases were searched from their respective inception dates up to the 18th May 2009: MEDLINE, Embase, PsychINFO, CINAHL, IBSS, AMED, BNI and Cochrane Review. Articles were included if they had a focus on spinal pain populations (search term keywords: back pain, low back pain, neck pain), measured informal social support (search term keywords: social support, social networks, family relations, social interaction) and provided data for the role of informal social support on association, risk or prognosis with spinal pain outcomes such as pain intensity, disability, recovery or associated psychological factors (search term keywords: risk factors, prospective studies, epidemiologic studies, cohort studies, cross-sectional studies, case-control studies). The search terms (Table S1, see the online version at 10.1016/j.ejpain.2010.09.011) were used as keywords and also exploded to include all lower level headings (e.g. Mesh terms within MEDLINE). Studies were excluded that focused on employment support, or included other health populations (e.g. cancer, diabetes), studies solely on pregnant women, studies of surgical cohorts (e.g. lumbar fusion patients), studies of back pain/neck pain patients who have a specific diagnosis (e.g. lumbar stenosis, spondylolithesis, spinal cord diseases, red flags) and small case series (e.g. studies of <30 people). Reference lists of the studies and current relevant reviews were checked for additional study citations. Validated measures of social support were also citation checked using the ISI Web of Science citation mapping system, and databases of local experts were consulted for information on additional research studies.

**Table S1:** Systematic review search strategy.

Term	Major headings	Keywords	Search text
*MEDLINE*			
Back pain	Back pain (exploded)	Back pain, backache, low back pain, sciatica, neck pain	(“Back pain”[Mesh] or “low back pain”[Mesh] or “sciatica”[Mesh] or “Neck pain”[Mesh] or “back pain”[Text Word] or “backache”[Text Word] or “sciatica”[Text Word] or “neck pain”[Text Word])
	Low back pain (exploded)		
	Sciatica (exploded)		
	Neck pain (exploded)		

Social support	Social support (exploded)	Family members, family member, kinship networks, kinship network, extended family, extended families, interpersonal relations, interpersonal relation, social interaction, interaction social, social interactions, interactions social, employee health services, occupational health services, employment support, employment based support	“Social support”[Mesh] or “social isolation”[Mesh] or “family relations”[Mesh] or “family members”[Text Word] or “family member”[Text Word] or “kinship network”[Text Word] or “kinship networks”[Text Word] or “extended family”[Text Word] or “extended families”[Text Word] or “interpersonal relations”[Text Word] or “social interaction”[Text Word] or “social interactions”[Text Word] or “interaction social”[Text Word] or “interactions social”[Text Word] or “employee health services”[Text Word] or “occupational health services”[Text Word] or “employment support”[Text Word] or “employment based support”[Text Word]
	Social isolation (exploded)		
	Family relations (exploded)		

Study Setting	Cohort studies (Exploded)		(“Cohort studies”[Mesh] OR “Epidemiologic studies”[Mesh] OR “Follow up studies”[Mesh] OR “Prospective studies”[Mesh] OR “Longitudinal studies”[Mesh] OR “Cross sectional studies”[Mesh] OR “Health surveys”[Mesh])
	Epidemiologic studies (exploded)		
	Follow-up studies (exploded)		
	Prospective studies (exploded)		
	Longitudinal studies (exploded)		
	Cross-sectional studies (exploded)		
	Health surveys (exploded)		


Term	Search text

*Cochrane*	
Back pain	Thesaurus and mesh tree search terms within title, abstract or keywords – back pain or back injuries or back pain with radiation or back pain without radiation or backache or low back pain or low back ache or low backache or mechanical low back pain or recurrent low back pain or postural low back pain or neck pain or sciatica

Social support	Thesaurus and mesh tree search terms within title, abstract or keywords – social support or social alienation or social isolation or social networks

Study settings	Thesaurus and mesh tree search terms within title, abstract or keywords – case-control studies or case-referent studies or case-control studies or cohort studies or closed cohort studies or historical cohort studies or correlation study or cross-sectional studies or cross-sectional studies or epidemiological studies or follow-up studies or follow-up studies or follow-up studies or incidence studies or longitudinal studies or matched case controlled studies or prevalence studies or prospective studies or retrospective study or epidemiologic studies

*AMED, IBSS and the British nursing index*	
Back pain	DE “back pain” or KW “neck pain” or KW “low back pain” or KW “sciatica” or AB “back pain” or AB “low back pain” or AB “sciatica” or AB “neck pain” or AB “lower back pain” or AB “lumbago” or AB “backache” or AB “back ache” or AB “lower back ache”

Social support	DE “social support” or DE “social networks” or DE “family relations” or DE “friendship” or DE “significant others” or DE “social interaction” or KW “social support” or KW “social networks” or AB “social support” or AB “social networks”

Study setting	(DE “between groups design” or DE “cohort analysis” or DE “follow-up studies” or DE “longitudinal studies” or DE “repeated measures” or DE “quantitative methods” or DE “mail surveys” or DE “telephone surveys”) or (TX “between groups design” or TX “cohort analysis” or TX “follow-up studies” or TX “longitudinal studies” or TX “repeated measures” or TX “quantitative methods” or TX “mail surveys” or TX “telephone surveys”)


Term	Major heading	Keyword	Search text

*PsychINFO*			
Back pain	Back pain (exploded)	Low back pain sciatica, backache, neck pain, lumbago, back ache, lower back pains, low back ache	DE “back pain” or KW “neck pain” or KW “low back pain” or KW “sciatica” or AB “back pain” or AB “low back pain” or AB “sciatica” or AB “neck pain” or AB “lower back pain” or AB “lumbago” or AB “backache” or AB “back ache” or AB “lower back ache”

Social support	Social support (exploded)	Social support, social networks	DE “social support” or DE “social networks” or DE “family relations” or DE “friendship” or DE “significant others” or DE “social interaction” or KW “social support” or KW “social networks” or AB “social support” or AB “social networks”
	Social networks (exploded)		
	Family relations (exploded)		
	Friendship (exploded)		
	Significant others (exploded)		
	Social interaction (exploded)		

Study setting	Between groups design (exploded or text terms)		(DE “between groups design” or DE “cohort analysis” or DE “follow-up studies” or DE “longitudinal studies” or DE “repeated measures” or DE “quantitative methods” or DE “mail surveys” or DE “telephone surveys”) or (TX “between groups design” or TX “cohort analysis” or TX “follow-up studies” or TX “longitudinal studies” or TX “repeated measures” or TX “quantitative methods” or TX “mail surveys” or TX “telephone surveys”)
	Cohort analysis (exploded or text terms)		
	Follow-up studies (exploded or text terms)		
	Mail surveys (exploded or text terms)		
	Telephone surveys (exploded or text terms)		
	Longitudinal studies (exploded or text terms)		


Term	Major heading	Search text

*Embase*		
Back pain	Backache (exploded)	(Back and pain or back and injuries or back and pain and with and radiation or back and pain and without and radiation or backache or low and back and pain or low and back and ache or low and backache or mechanical and low and back and pain or recurrent and low and back and pain or postural and low and back and pain or neck and pain or sciatica or lumbago or lumbalgesia or lumbal and pain or lumbar and pain or lumbalgia or lumbosacral and pain).ti,ab or (exp Backache/)

Social support	Social support (exploded)	(exp *social network/ or exp FAMILY/ or exp Social Structure/ or exp social support/ or exp social interaction/)
	Social network (exploded)	
	Family (exploded)	
	Social structure (exploded)	
	Social interaction (exploded)	

Study setting	Longitudinal study (exploded)	exp longitudinal study/ or exp follow up/ or exp case-control study/ or exp cross-sectional study/ or exp cohort analysis/ or exp epidemiology/ or exp prevalence/ or exp questionnaire/)
	Follow up study (exploded)	
	Case control study (exploded)	
	Cross-sectional study (exploded)	
	Cohort analysis (exploded)	
	Epidemiology (exploded)	
	Prevalence (exploded)	
	Questionnaire (exploded)	


Term	Major headings	Keywords	Search text

*CINAHL*			
Back pain	Back pain low back pain, sciatica	Back pain, low back pain, backache, back ache, sciatica, neck pain, lumbago	(MH “back pain+”) or (MH “lower back pain”) or (MH “back”) or (MH “sciatica”) or (MH “neck pain”) or (“lumbago”) or (“lower back pain”) or (“back pain”) or (“neck pain”) or (“backache”) or (“backache”)
	Neck pain		

Social support	Social support	Social support social networks emotional support family support	(“social support”) or (MH “Norbeck social support questionnaire”) or (MH “social support (Iowa NOC)”) or (MH “social support index”) or (MH “support, psychosocial+”) or (“social networks”) or (MH “social networks”) or (“emotional support”) or (“family support”) or (MH “family support (Iowa NIC)”) or (MH “extended family”) or (MH “social support (Iowa NOC)”)
	Norbeck social support questionnaire		
	Social support Iowa NOC		
	Social support index		
	Support, psychosocial (exploded)		
	Social networks		
	Family support (Iowa NIC)		
	Extended family		

Study settings	Experimental studies		(MH “experimental studies”) or (MH “nonexperimental studies”) or (MH “concurrent prospective studies”) or (MH “cross-sectional studies”) or (MH “health policy studies”)
	Nonexperimental studies		
	Concurrent prospective studies		
	Cross-sectional studies		
	Health policy studies		

### 2.2 Article quality assessment

It was not possible to use a pre-existing quality assessment tool to assess article quality due to the inclusion of differing study designs (e.g. cohort, cross-sectional) and so the quality assessment measure (Table S2, see the online version at 10.1016/j.ejpain.2010.09.011) was based on the combination of assessments of a number of recent review articles and guidance on quality assessment within systematic reviews on the area of back pain (Hoogendoorn et al., 2000, Woods, 2005, Mallen et al., 2007, Hayden et al., 2008, Lakke et al., 2009). Article quality was assessed by considering the following components: having a clear research objective, describing the recruitment procedure, describing the inclusion exclusion criteria, describing the population parameters/demographics, describing participation rates, describing the measure of social support, reporting the strength of effect, use of multivariate analysis, having an adequate sample size, acknowledging the limitations of their research, and reporting a participation rate above 70%. For cohort studies three further criteria applied in the assessment of article quality: the reporting of attrition rates and information on responders and non responders, an attrition rate below 20% and a follow up time period over 6 months. Taking into account the reported lack of studies on informal social support, within spinal pain populations, the authors decided that there would be no exclusions from the quality assessment. Articles were assessed using the quality assessment criteria checklist by two reviewers (GW, PC). Thereafter all disagreements were discussed at a consensus meeting and if disagreements were not resolved, a third reviewer (KMD) provided the final judgement.

**Table S2:** Quality assessment table for included studies.

### 2.3 Data extraction

Study information on author, country, study population, sample size, response rate, follow up period (cohort designs only), study design, focus, assessment of spinal pain, assessment of social support, analysis, outcome in relation to social support, findings and strength of reported effect were extracted from the studies.

### 2.4 Analysis

In order to meaningfully apply the information on article quality to assist in the interpretation of the results (e.g. high quality studies having more weight than a low quality studies) the authors decided to use tertiles (three equal sized groups) to create quality score categories for the included studies: ‘high’, ‘medium’ and ‘low’ quality. A best evidence synthesis was carried out to assess the weight of evidence (Slavin, 1995) using levels of evidence criteria adapted from guidance on qualitative synthesis for randomised controlled trials (RCTs) (van Tulder et al., 2003), and subsequent development for non RCT designs (Licht-Strunk et al., 2007). Table 1 outlines the criteria for the assessment of evidence. To overcome the issue of heterogeneity, studies were combined on study design (occurrence, prognosis, cross-section) and type of social support (emotional, instrumental, informational, appraisal, network size, frequency of support and satisfaction).

**Table 1:** Levels of evidence for associations of informal social support and spinal pain.

Level of evidence	
*Statistical significant associations*	
Strong	Consistent associations found in at least two high quality studies
Moderate	Consistent associations found in one high quality study and at least one medium or two low quality studies
Weak	Associations found in at least two medium or three low quality studies
Inconclusive	Associations found in less than three medium/low quality studies
Inconsistent	Inconsistent findings irrespective of study quality

*Associations without statistical significance*	
Inconclusive	No significant association found in at least two studies
Insufficient	Only one study presenting no statistical association, irrespective of study quality

## 3 Results

### 3.1 Literature search

**Fig. 1:**
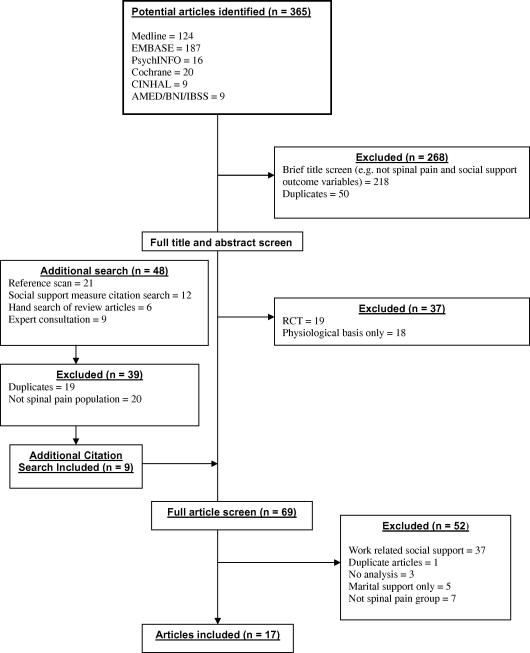
Flow diagram of identification and inclusion of papers for review.

The systematic search using the databases resulted in 365 publications (see Fig. 1 for a flow diagram of the review procedure). A further 48 articles were included via additional search strategies (hand search, expert consultation, citation search). Three hundred and fourty-four articles were excluded at the title and abstract screen search stage with a further 52 articles excluded using full text screening. The reasons for exclusion at the full text screening stage were studies solely focusing on employment support, studies on specific spinal pain populations (e.g. spondylolithesis, lumbar stenosis), or populations that focused on chronic pain patients outside of this study’s inclusion criteria (e.g. migraines, fibromyalgia, chronic widespread pain). This resulted in 17 suitable articles included within the review (Blozik et al., 2009, Feleus et al., 2007, Follick et al., 1985, Hurwitz et al., 2006, Isacsson et al., 1995, Khatun et al., 2004, Klapow et al., 1995, Koleck et al., 2006, Larsen and Leboeuf-Yde, 2006, Linton, 2005, Masters et al., 2007, Muramatsu et al., 1997, Power et al., 2001, Schneider et al., 2005, Skov et al., 1996, Takeyachi et al., 2003, Trief et al., 1995). One study (Muramatsu et al., 1997) reported both on prospective cohort results for occurrence and also on follow up results for prognosis and will therefore be used in both occurrence and prognosis sections of the analysis.

### 3.2 Quality assessment analysis

Studies with a score below 73 were classified as low quality (*n* = 5), a score between 73 and 91 as medium quality (*n* = 7) and a score above 91 as high quality (*n* = 5). All studies offered a clear research objective, all but one study described their recruitment procedure adequately, 13 studies gave descriptions of their inclusion/exclusion criteria, all but one study described the demographics of their study populations and 12 studies reported participation rates at baseline, but only one third of these reached a quality target criteria of 70% participation rate. For the cohort designs, three studies report a follow up period of 3 years or more (Khatun et al., 2004, Muramatsu et al., 1997, Power et al., 2001), one study reports a follow up of 12 months (Koleck et al., 2006), one study reports a six month follow up period (Hurwitz et al., 2006) and one study reports a 3 month follow up period (Larsen and Leboeuf-Yde, 2006). Cohort studies had the greatest combined level of quality (88%) compared to cross-sectional studies (74%). Full descriptive data extraction tables can be found online (Table S3, Table S4, Table S5, see the online version at 10.1016/j.ejpain.2010.09.011). A summary table of study findings and study quality can be found below in Table 2.

**Table 2:** Summary of findings on associations of informal social support and spinal pain.

Outcome	Study	Area of assessment	Type of support	Evidence of effect	Study quality
Occurrence	Khatun et al.	Neck and back	Emotional	+ (females only)	High
	Khatun et al.	Neck and back	Network	×	High
	Larsen et al.	LBP	Network	×	Medium
	Linton et al.	LBP	Not specified	×	Medium
	Muramatsu et al.	LBP	Instrumental	+	High
	Muramatsu et al.	LBP	Emotional	−	High
	Power et al.	LBP	Emotional	×	High
	Power et al.	LBP	Instrumental support	×	High

Prognosis	Hurwitz et al.	Neck pain	Emotional	+ (reduction in pain over time)	High
	Hurwitz et al.	Neck pain	Emotional	× (disability)	High
	Hurwitz et al.	Neck pain	Instrumental	+ (reduction in disability over time)	High
	Hurwitz et al.	Neck pain	Instrumental	× (pain)	High
	Koleck et al.	LBP	Network	× (recovery)	Low
	Muramatsu et al.	LBP	Instrumental	× (recovery)	High
	Muramatsu et al.	LBP	Emotional	− (decrease in recovery)	High

Cross-section (spinal pain outcomes)	Blozik et al.	Neck pain	Global	×	Medium
	Isacsson et al.	Neck and back	Instrumental	+	High
	Isacsson et al.	Neck and back	Network	+	High
	Isacsson et al.	Neck and back	Frequency	×	High
	Isacsson et al.	Neck and back	Emotional	×	High
	Schneider et al.	LBP	Network	×	Medium
	Skov et al.	Neck and back	Network	×	Medium
	Takeyachi et al.	LBP	Network	×	Low
	Takeyachi et al.	LBP	Frequency	×	Low

Cross-section (psychological outcomes)	Feleus et al.	Neck pain	Satisfaction/network	+ (kinesiophobia)	High
	Follick et al.	LBP	Social interaction	+ (MMPI variables)	Low
	Klapow et al.	LBP	Satisfaction	+ (low depression)	Low
	Masters et al.	LBP	Satisfaction	+ (catastrophising)	Low
	Trief et al.	LBP	Satisfaction/network	+ (low depression)	Medium

LBP (low back pain), + (significant positive effect), − (significant negative effect), × (no significant effect), MMPI (Minnesota Multiphasic Personality Inventory).

**Table S3:** Summary of cross-sectional studies on informal social support and spinal pain outcomes.

Author (Year)	Country	Study population (*N*=)	Quality score (%)	Main study focus	Assessment spinal pain	Assessment social support	Analysis (adjusted or univariate)	Study outcome	Findings	Effect
*Cross-sectional associations with pain outcomes*										
Blozik et al. (2009)	Germany	448 (38%) Primary care sample (neck pain consulters)	91	Depression and anxiety as determinants of neck pain	Neck pain and disability scale (20 item measure of neck pain severity and related disability)	Sarason Social Support Questionnaire (adapted 14 item)	Linear regression (adjusted)	Neck pain scale score	Adjusted regression analysis showed no significant association of social support on neck pain	N/S

Isacsson et al. (1995)	Sweden	500 (80%) Participants in cohort study of men born in Malmo, Sweden	100	Prevalence of neck and back pain	Self rate musculoskeletal disability questionnaire on neck and back pain in previous 12 months	Comprehensive model including social network and frequency of contact, participation in social activities, emotional support, material support, satisfaction with support	Logistic regression (adjusted)	Prevalence of neck and back pain	A significantly greater risk of back/neck pain was associated with lower levels of instrumental social support	OR 1.6 (1.0–2.7)
									A significant association was reported on social anchorage and back/neck pain	OR 2.1 (1.2–3.6)
									There was no significant associations between frequency of contact with network or emotional support and back pain	N/S

Schneider et al. (2005)	Germany	3488 (61%) Sample of the working population	82	The role of workplace, lifestyle and social factors on back pain	Prevalence of back pain within previous 7 days	Number of people within network that can be depended on	Multiple regression. Further analysis based on gender	Relationship between back pain and gender	No significant relationship was reported with social support and back pain for both men and women	N/S

Skov et al. (1996)	Denmark	1306 (66%) Random sample of salespeople within Association of Danish Active Salespeople	73	Physical and psychosocial risk factors of back, neck and shoulder pain	Nordic questionnaire on pain intensity previous back pain over past 12 months	Social network	Logistic regression (adjusted)	Symptoms of neck, shoulder and back pain	Social network was not entered into the final analysis for psychosocial risk factors	N/S

Takeyachi et al. (2003)	Japan	816 (98%) Patients attending a medical examination	55	Assessment of correlations among back pain outcome measures	Presence of LBP within previous 24 h VAS pain intensity	Social network size and frequency of interaction	Path analysis (adjusted)	LBP status and severity	No association with back pain VAS scores and social network	N/S
									No association with frequency of social interaction and back pain	N/S

*Cross-sectional associations with psychological outcomes*										
Feleus et al. (2007)	Netherlands	679 (85%) baseline	100	Kinesiophobia in relation to arm, neck, shoulder pain	Disability of arm, shoulder and hand (DASH) questionnaire	Social support scale (adapted Sarason SSQ)	Multiple regression of cross-sectional data at baseline (adjusted)	Kinesiophobia score at baseline	Univariate analysis showed effect of social support on levels of kinesiophobia at baseline	*β* – 2.33 (1.37–3.29) *p* < 0.1
					Pain severity scale				Multivariate regression analysis retained social support within the model as a factor contributing to kinesiophobia. Total model accounted for 24% of the variance in kinesiophobia	*β* – 1.17 (0.28–2.05)
		Patients consulting GPs for neck, back, elbow, wrist or arm pain			Manikin					

Follick et al. (1985)	USA	107 Participants with CLBP referred to a chronic pain treatment programme	55	Disability and emotional levels within the CLBP population	Medically assessed as part of referral to treatment programme with primary complaint of CLBP	Subscale of the sickness impact profile on social interaction	Correlation (crude)	Correlations between psychosocial factors and the MMPI	Psychosocial factors, inclusive of social interaction variable, correlated significantly with all MMPI variables	Reported associations with MMPI subscales (*p* < 0.01) as part of a psychosocial dimension

Klapow et al. (1995)	USA	95 Consecutive male patients with CLBP recruited from a general orthopaedic clinic	55	Linkage of psychosocial variables with clinical subgroups of back pain	Previously validated sub groups of back pain	Sarason Social Support Questionnaire (satisfaction scale only)	Discriminant function analysis (DFA)	Differences in the level of social support between sub groups	Sub group with highest pain level and highest depression scores had significantly lower levels of social support than other two sub groups	ANOVA
							ANOVA (univariate)			*F*(2, 92) 10.32, *p *< 0.01

Masters et al. (2007)	USA	50 Consecutive patients attending spine rehabilitation clinic	55	Patient perceptions of social support	Referral and attendance at spine clinic	Quality ratings of received social support from family, friends, spouse and formal levels	Comparison test (univariate)	Relationship to catastrophising subgroups (low/high)	Those with higher levels of catastrophising reported lower levels of instrumental support	*X^2^* (20, *N* = 48) = 33.93, *p = *0.03

Trief et al. (1995)	USA	70 Patients with chronic back pain (>6 months) attending a rehabilitation programme	82	The role of social support within a depression/chronic pain model	Medical assessment as entry criteria to rehabilitation programme	Sarason Social Support Questionnaire (network and satisfaction)	Comparison between groups stratified on depression scores (univariate)	Levels of depressive symptoms	Non depressed back pain patients found to have significantly more people offering support	*t* = 2.04, *p *< 0.05
									Non depressed back pain patients rated the quality of the social support that they receive as higher compared to depressed group	*t* = 3.02, *p *< 0.004

CLBP – chronic low back pain, LBP – low back pain, *β* – beta, OR – Odds Ratio, ANOVA – Analysis of variance, N/S – not significant, VAS – visual analogue scale.

**Table S4:** Summary of cohort studies on informal social support and occurrence of spinal pain.

Author (year)	Country	Study population (follow up period)	Quality score (%)	Main study focus	Assessment spinal pain	Assessment social support	Analysis (adjusted or univariate)	Study outcome	Findings	Effect
*Cohort studies on occurrence of spinal pain*										
Khatun et al. (2004)	Sweden	1083 (100%)	93	Social class and other factors within childhood and adolescence in relation to musculoskeletal (MSD) disorders	Presence of pain in hips, back, shoulder, neck, hands, elbows, knees	Social network (number of persons)	Logistic regression (univariate)	Risk of MSD	There was no significant association reported for social support or network and risk of MSD in men	
		1044 (96%)			Indication of pain severity (non, mild, severe)	Social support (emotional and material)			There was no significant effect of social network and risk of MSD in women	N/S
		Follow up							There was a small significant effect of social support and risk of MSD for women	N/S
		School leavers (14 year follow up)								OR 1.09 (1.0–1.19)

Larsen and Leboeuf-Yde (2006)	Denmark	357 (98%) baseline	86	Association with coping and LBP	Leg and/or back pain in previous 3 months	Single question on level of support from friends/family	Logistic regression (univariate)	Incidence of back pain	Level of support from family had no significant effect on back pain incidence	N/S
		331 (92%) follow up			Discharge from duties due to back pain					
		Military conscripts (3 month follow up)								

Linton (2005)	Sweden	1914 (69%)	86	Risk of back pain from psychosocial factors using cross-sectional and prospective analysis	Self report back pain in previous 12 months with questions on worse, average and frequency of pain	Undefined	Logistic regression (adjusted)	Risk of prevalent or incident back pain		

Muramatsu et al. (1997)	Japan	Random population sample (12 month follow up)		Sociomedical perspective on the transitions in CLBP	Self report question on current experience of LBP (Time 1). Self report question on experience of chronic LBP (Time 2)	Emotional and instrumental support from a significant other	Logistic and multiple regression (adjusted)	Onset of LBP (Time 1)	No significant contribution reported of social support on risk of back pain	N/S
		2200 (68%)	93					Recovery of LBP (Time 2)	Instrumental support reduced risk of back pain from no pain (T1) to back pain (T2)	
		1986 (90%) Follow up							Emotional support increased risk of back pain from no pain (T1) to back pain (T2)	−2.25%
		General sample of over 60 population (3 year follow up)								1.17%

Power et al. (2001)	UK	5871 (50%) baseline	93	Predictors of LBP	Self report questions on presence of back pain	six social support questions (emotional and practical) from the British social attitudes survey	Logistic regression (univariate)	Incidence of LBP	No significant relationship found for emotional social support and incidence of back pain	N/S
		5871 (100%) follow up			Manikin				No significant relationship found for practical social support and incidence of back pain	N/S
		Birth cohort								

CLBP – chronic low back pain, LBP – Low back pain, β – beta, OR – Odds Ratio, ANOVA – analysis of variance, N/S – not significant, VAS – visual analogue scale.

**Table S5:** Summary of cohort studies on informal social support and prognosis of spinal pain.

Author (year)	Country	Study population (follow up period)	Quality score (%)	Main study focus	Assessment spinal pain	Assessment social support	Analysis (adjusted or univariate)	Study outcome	Findings	Effect
*Cohort studies on prognosis for spinal pain outcome*										
Hurwitz et al. (2006)	USA		91	Impact of psychosocial variables on neck pain and associated disability		Four questions on instrumental and emotional support and frequency of support	Linear regression (adjusted)			
		Secondary analysis of RCT			Neck disability index			2 + Point reduction in severe and average pain	Greater emotional support was shown to have significant effect on average pain reduction	OR 2.26 (1.03–4.95)N/S
		336 (35%) baseline			Pain rating for average and severe pain			5 + Point reduction in neck disability score at 6 months	There was no significant effect of emotional support on severe pain or neck disability	OR 2.94
		268 (79%) Follow up							Higher instrumental support was associated with a reduction in neck disability	(1.32–6.58)
		Health care population invited to take part in RCT (6 month follow up)							There was no effect of instrumental support on pain levels (severe/average)	N/S

Koleck et al. (2006)	France		64	Coping strategies in those with LBP	Self report LBP	Perceived social support scale (adapted from the Sarason SSQ including quality and availability of support)	Principal components analysis (adjusted)	Assessment of acute (T1) to chronic stage (T2). PCA to form factors influencing outcome	Social support quality was dropped from initial analysis	Not reported
		99 Baseline							Social support availability remained in PCA model as part of ‘perceived control’ factor. Perceived control did not contribute to improvement over time	N/S
		90 (90%) Follow up			VAS pain intensity					
		Consecutive GP consulters with new episode of nonspecific back pain (12 month follow up)								

Muramatsu et al. (1997)	Japan	2200 (68%)	93	Sociomedical perspective on the transitions in CLBP	Self report question on current experience of LBP (Time 1). Self report question on experience of chronic LBP (Time 2)	Emotional and instrumental support from a significant other	Logistic and multiple regression (adjusted)	Onset of LBP (Time 1)	Instrumental and emotional support did not reduce chronic status (back pain at T1 and back pain at T2).	N/S
		1986 (90%) Follow up						Recovery of LBP (Time 2)	Instrumental support did not significantly contribute to recovery (back pain T1 to no back pain T2).	N/S
		General sample of over 60 population (3 year follow up)							Emotional support decreased recovery status (back pain T1 to no back pain T2).	−2.93%

CLBP – chronic low back pain, LBP – low back pain, β – beta, OR – Odds Ratio, ANOVA – analysis of variance, N/S – not significant, VAS – visual analogue scale.

### 3.3 Study assessment of social support and spinal pain

The Sarason Social Support Questionnaire (SSSQ, Sarason et al., 1983) or an adapted version was chosen by five studies (Blozik et al., 2009, Feleus et al., 2007, Klapow et al., 1995, Koleck et al., 2006, Trief et al., 1995). The SSSQ measures the constructs of network size and perceived satisfaction for emotional support. A further 11 studies employed various social support measures that measured different aspects of informal social support: network size (Isacsson et al., 1995, Khatun et al., 2004, Larsen and Leboeuf-Yde, 2006, Schneider et al., 2005, Skov et al., 1996, Takeyachi et al., 2003), frequency of support (Follick et al., 1985, Hurwitz et al., 2006, Isacsson et al., 1995, Takeyachi et al., 2003), satisfaction with support (Isacsson et al., 1995, Masters et al., 2007), emotional support (Hurwitz et al., 2006, Isacsson et al., 1995, Muramatsu et al., 1997, Power et al., 2001), and instrumental support (Isacsson et al., 1995, Muramatsu et al., 1997, Power et al., 2001). One study offered no description of their measure of social support (Linton, 2005). Studies reported variation on the time scale for the assessment of spinal pain, with one study using the presence of pain within a previous 24 h period (Takeyachi et al., 2003), one in the previous 7 days (Schneider et al., 2005), one in the previous 3 months (Larsen and Leboeuf-Yde, 2006), four within the previous 12 months (Isacsson et al., 1995, Linton, 2005, Muramatsu et al., 1997, Skov et al., 1996) with a further six studies having no specified time period within their articles (Blozik et al., 2009, Feleus et al., 2007, Hurwitz et al., 2006, Khatun et al., 2004, Koleck et al., 2006, Power et al., 2001). Other studies based their assessment of spinal pain on medical assessment or attendance at a spinal pain clinic (Follick et al., 1985, Masters et al., 2007, Trief et al., 1995) or absence from work (Larsen and Leboeuf-Yde, 2006). In addition to the measure of the presence of pain, eight studies (Blozik et al., 2009, Feleus et al., 2007, Hurwitz et al., 2006, Khatun et al., 2004, Koleck et al., 2006, Linton, 2005, Skov et al., 1996, Takeyachi et al., 2003) reported the use of a pain intensity measure (e.g. visual analogue scale), a further five studies included a measure of disability (Blozik et al., 2009, Feleus et al., 2007, Follick et al., 1985, Hurwitz et al., 2006, Isacsson et al., 1995).

### 3.4 Cross-sectional associations with informal social support

There are five studies, one of high quality (Isacsson et al., 1995), three of medium quality (Blozik et al., 2009, Schneider et al., 2005, Skov et al., 1996) and one of low quality (Takeyachi et al., 2003), that use cross-sectional designs and report the association of informal social support on pain (see Table S3). For emotional support, only one high quality study (Isacsson et al.) reports the association of emotional support and neck pain. The study reports no significant association, and best evidence synthesis indicates that there is insufficient evidence to reach a conclusion. One study (Isacsson et al.), reports on instrumental support, with a significant finding of lower levels of instrumental support being associated with higher risk of back and neck pain (Odds Ratio, OR – 1.6). Best evidence synthesis indicates a weak level of evidence for the association between instrumental support and spinal pain in a cross-sectional design. Five studies report the association between social network size and spinal pain. One high quality study (Isacsson et al.) reports that higher levels of social anchorage (a measure of social network) are associated with lower risk of neck and back pain (OR 2.1). Three medium quality studies (Blozik et al., Schneider et al., Skov et al.) and one low quality study (Takeyachi et al.) report no effect. Best evidence synthesis indicates inconclusive evidence of the association between network size and pain within cross-sectional designs. Two studies report the association between frequency of contact with those who offer social support and spinal pain. One high quality (Isacsson et al.) and one low quality study (Takeyachi et al.) report no significant association. Best evidence synthesis indicates inconclusive evidence of an association between frequency of contact on pain. No studies within this group reported on the association between appraisal, informational support or satisfaction with social support.

One high quality study (Feleus et al., 2007), one medium quality (Trief et al., 1995) and three low quality studies (Follick et al., 1985, Klapow et al., 1995 and Masters et al., 2007), report on the association of informal social support with psychological factors (e.g. depression, kinesiophobia, catastrophising). Four studies, one high quality (Feleus et al.), one medium (Trief et al.) and two low quality (Klapow et al., Masters et al.) all stratified groups of spinal pain patients dependent on psychological outcomes, and all report significant group differences, with those more severely affected by psychological outcome having lower levels of satisfaction with social support. Best evidence synthesis indicates moderate evidence of an association between satisfaction with social support and psychological outcomes in patients with nonspecific spinal pain. Frequency of interaction with social support and psychological outcome is reported by one low quality study (Follick et al.). The study reports that social interaction correlates with psychological scales of the Minnesota Multiphasic Personality Inventory (MMPI). Best evidence synthesis indicates inconclusive evidence on the association between frequency of interaction and psychological outcomes. No studies reported associations with emotional, instrumental or informational support, appraisal or network size.

### 3.5 Informal social support and occurrence of spinal pain

Five cohort studies, three of high quality (Khatun et al., 2004, Muramatsu et al., 1997, Power et al., 2001) and two of medium quality (Larsen and Leboeuf-Yde, 2006, Linton, 2005), considered informal social support and the occurrence of spinal pain (see Table S4). Three high quality studies (Khatun et al., Muramatsu et al., Power et al.) report the association between emotional social support and occurrence of spinal pain. Khatun et al. reports of a small association for females with neck pain, Power et al. reports no effect for back pain and Muramatsu et al. report a small inverse effect with emotional support increasing risk of back pain. Best evidence synthesis indicates inconclusive evidence of an effect of emotional support on risk of spinal pain. Two high quality studies (Muramatsu et al., Power et al.) report on the effects of instrumental support. Muramatsu et al. report on a slight decrease (2%) in risk of low back pain with higher instrumental support, and Power et al. report no significant effect. Best evidence synthesis indicates inconsistent findings for the effect of instrumental support on spinal pain. Two studies, one high quality (Khatun et al.) and one medium quality (Larsen and Leboeuf-Yde) report the effects of social network size from friends and family and risk of spinal pain. Both studies report no significant associations, indicating inconclusive evidence using best evidence synthesis. One medium quality study (Linton et al.) failed to define their measure of social support and so cannot be included within the best evidence synthesis, but the direction of their result shows no evidence of an effect. No further studies reported on informational support, appraisal, satisfaction or frequency of interaction with social support.

### 3.6 Informal social support and prognosis of spinal pain

Three cohort studies considered the effect of social support on outcome over time within spinal pain populations (Hurwitz et al., 2006, Koleck et al., 2006, Muramatsu et al., 1997) (see Table S5). One high quality (Muramatsu et al.) and one medium quality (Hurwitz et al.) report the effect of emotional support on prognosis. Hurwitz et al. report higher levels of emotional support related to lower average ratings of neck pain (OR 2.26), but no effects for disability. However, Muramatsu et al. report that emotional support increased the recovery time for those with back pain. Best evidence synthesis suggests inconsistent evidence of an effect of emotional support on prognosis for those with spinal pain. Both Hurwitz et al. and Muramatsu et al. report the effects of instrumental support (e.g. counting on someone with help for daily tasks or when ill) on prognosis. Hurwitz et al. report higher levels of instrumental support relating to lower levels of neck disability (OR 2.94), but no effect for instrumental support on pain severity. Muramatsu et al. report no significant effect of instrumental support on recovery status or lowering pain. Best evidence synthesis indicates inconsistent evidence of an effect of instrumental support on prognosis for those with spinal pain. One low quality study (Koleck et al.) reports satisfaction with support, and size of network available to offer support, in association with acute to chronic stages, for those with low back pain. In both results, Koleck et al. report no significant findings, and according to best evidence synthesis there is insufficient evidence to draw any conclusion. No further studies reported effects for the association of informational support, appraisal and frequency of support.

## 4 Discussion

This review considered the evidence on the effects of informal social support on two epidemiological aspects of spinal pain. Firstly the review considered evidence of occurrence, in effect does the level or type of informal support a person has influence the risk of developing spinal pain. Secondly the review looked at evidence of an effect of social support on prognosis, considering aspects such as pain reduction and recovery. In addition the review has also summarised the contribution of informal social support on the psychological aspects in patients with spinal pain. The results on occurrence and prognosis for pain outcome (e.g. pain severity, recovery, disability) are on the whole inconsistent and inconclusive. However the review reports that in cross-sectional studies, social support was more associated with psychological factors related to pain outcome than to pain, which could be suggestive that informal social support may influence outcome indirectly, by moderating psychological factors associated with spinal pain.

There are two main theoretical models that have been put forward to explain the effects of social support (Chronister et al., 2006). The first is the direct or ‘*main*’ effect model whereby it is thought that having greater levels of social support promotes general good health and therefore less risk of developing illness. The second model is the ‘*stress buffering*’ model whereby social support acts to alleviate and reduce stress, which then lessons the chance of illness or speeds recovery after adversity. In view of this reviews’ findings on the association between informal social support and psychological outcomes and lack of findings on risk there appears to be greater supportive evidence of the latter model. The evidence from the association of informal social support and psychological outcome suggests that those with spinal pain who report greater detrimental psychological outcomes (e.g. greater catastrophising, greater kinesiophobia and greater depression) also report lower levels of informal support. It is well established that psychological factors have been shown to play an important part on the prognosis associated with spinal pain (Keefe et al., 2004, Pincus et al., 2002). The level and type of informal social support may be an important factor for psychological well-being and this may have a moderating effect between psychological outcomes and spinal pain. However most of the studies that considered these associations within this review are low quality, have small sample sizes, report univariate findings and are cross-sectional in design. Consequently it is difficult to ascertain whether social support influences psychological reactions to pain or vice versa. Furthermore studies using univariate analysis failed to adjust for the variation effect of pain intensity which has been shown to have strong associations with psychological outcomes such as depression (Keefe et al., 2004).

Considering the findings on occurrence and prognosis from longitudinal cohort designs, the results on the influence of informal social support are inconclusive, inconsistent or insufficient. This is mainly due to the low number of studies that can be included within anyone analysis group, for example the association between satisfaction of support and prognosis was only reported by one study and so no synthesis could be made. Nevertheless, taking an overall view for risk of occurrence, of nine reported findings from the five studies, only two studies reported minor significant effects, suggesting that overall social support is unlikely to be a risk factor for spinal pain. For prognosis, of the three studies reporting nine findings, two of those findings were insufficient due to having only one study and a further four findings were inconsistent but the significant effects were larger than those reported for occurrence (OR > 2) suggesting more evidence is needed. Interestingly studies on neck pain appeared to report the clearest evidence of an effect, with Khatun et al. reporting a weak association of emotional support and risk of neck pain for females and Hurwitz et al. reporting effects for instrumental and emotional social support on reductions in disability and reductions in average pain severity. Evidence shows that compared to back pain there is a lower prevalence and incidence of neck pain, less disability is associated with neck pain and the life time trajectory of neck pain is thought to be more episodic (Guzman et al., 2008). It may that when a person gets neck pain, the help and assistance they receive has more impact due to these differences. However considering the two papers that report an effect, Hurwitz et al.’s sample consisted of those who were entered into an RCT from a clinical setting, and Khatun et al.’s finding is of an effect is only reported for females. This may limit the generalisibility in comparison to population level studies.

There are also other factors of heterogeneity that may have influenced the findings of this review. For example two studies (Isacsson et al. and Muramatsu et al.) both report significant findings on instrumental support and the reduction of risk in back and neck pain. However, both cohorts are of people over the age of 60. Research does suggest that with increasing age there is increasing chance of ill health and a greater need of support from family and friends (Trouillet et al., 2009). It may well be that social support has more of an effect for older persons who experience spinal pain. Another issue is time scale of assessment of spinal pain, with some of the cross-sectional studies having assessed spinal pain over shorter time periods than others. For example the presence of spinal pain at the time of the study or in the previous 24 h compared to the presence of spinal pain in the past 12 months. This has consequences in terms of comparing acute and chronic pain cohorts, with the former more likely to recover (Dunn et al., 2006, Chou et al., 2007). More importantly, as Waddell (2004) describes on social effects for back pain, the initial reaction of family and friends, when a person first gets back pain will be to rally round, but after a few weeks this support may diminish and therefore support for those with chronic back pain may differ from those at the acute stages.

There are also difficulties in the measurement of social support with many different measures and constructs used by the articles included within this review. Evidence does suggest there are difficulties in the conceptualisation and measurement of social support (Hutchison, 1999, Chronister et al., 2006). Additionally the only other review, we are aware of, that has a focus on informal social support in relation to spinal pain (in this case back pain) state there is insufficient evidence based on a considerable heterogeneity in the measurement and conceptualisation of social support within those studies (Hoogendoorn et al., 2000). It is important that future studies report what measures they employ and what social support factor, or factors, are measured to give a full assessment of the structure of social network (e.g. family, friends, other), the size of the network (e.g. number of people who offer support), the type of support offered (emotional, instrumental, information, appraisal) and the rating of satisfaction for the support (perceived support) so that future synthesis is possible.

The search strategy used in this review was comprehensive, with a wide-ranging search of electronic databases, supplemented by hand-searches of cited literature, reference lists and local databases. However, the review only included studies written in English within peer reviewed journals, and so may have missed important findings from other sources (grey literature). The method of quality assessment has advantages in terms of using a best evidence synthesis. The synthesis gives structure to the assessment of the included articles and also addresses some of the issues of heterogeneity outlined by Hoogendoorn et al.’s previous review. One disadvantage of this, within this review, is that only a few articles could be compared for each category (e.g. type of support) leading to conclusions of inconsistency. There is also the issue of quality assessment, in that study quality was assessed as a whole for each study, but many lower quality studies employed better measures of social support.

In terms of clinical relevance, the overall picture suggests that informal social support may be an important factor in the psychological well-being of the person with spinal pain, but the evidence is generally inconclusive. Furthermore, although speculative, the evidence does suggest there may be greater relevance of informal social support effects for older persons with spinal pain and that there may be greater effects for those with neck pain, but further research is needed.

### 4.1 Conclusion

This review has shown that there is inconclusive evidence of an effect of informal social support on the risk of occurrence of spinal pain. Evidence on prognosis is inconsistent and more research is required before conclusions can be made. Cross-sectional findings show a weak effect for instrumental support and pain and moderate evidence of an effect of satisfaction with the level of informal social support and psychological outcomes. More research is needed fully understand the influence of informal social support on nonspecific spinal pain using measures that encompass the complex dimensions of informal social support.
